# A distinct look at a transcendental phenomenon: the grounded theory model of leader humour

**DOI:** 10.1186/s40359-025-02511-8

**Published:** 2025-03-18

**Authors:** Mohammad Gholami, Fariborz Rahimnia, Gholamreza Malekzadeh, Alireza Khorakian

**Affiliations:** 1 Department of Islamic Management, Institute of Islamic Science in Humanities, Ferdowsi University of Mashhad, Mashhad, Iran; 2 Department of Management, Faculty of Economics and Administrative Sciences, Ferdowsi University of Mashhad, Mashhad, Iran

**Keywords:** Sense of humour, Humour (Humor), Leadership, Grounded theory (GT), Education

## Abstract

**Background:**

Humour is an essential quality and key factor in communication, particularly in leadership. This study explores leader humour within university departments to design a comprehensive model using grounded theory. The study also examines the effects and dynamics of humour in leadership and its influence on followers.

**Methods:**

This qualitative study employed Glaser’s 6 C family approach. Data were collected from 18 Iranian university professors, selected via purposive sampling until theoretical saturation was reached. Semi-structured interviews were conducted, and data were analysed using MAXQDA2020 software to facilitate the coding process.

**Results:**

The axial category was named ‘leader humorous behaviour’ and the main attributes of humour were ‘benign violation’ and ‘moderation’. This study highlights the importance of moderation in humorous behaviour (frequency and repetition) as an important attribute of leader humour alongside benign violation, which can have negative and unwanted outcomes for both leader and followers despite positive and constructive content. The final model identifies a sense of humour as the central cause and organisational factors as correlated causes. It also identifies the mediators, moderators, context, and consequences of leader humorous behaviour. Sense of humour is found to be the most important factor in followers’ perception and interpretation of leader humour.

**Conclusions:**

Leader humour can have positive ethical implications in organisations, thus enhancing relationships and communication when employed appropriately. The findings suggest that positive outcomes of leader humour over time foster greater expression and mitigate misunderstandings. This study offers a foundational understanding of leader humorous behaviour and its potential positive outcomes in organisational settings.

## Introduction

Happiness is an important aspect of a university environment, and low levels of happiness among university students have been reported worldwide and have received considerable attention [1]. Recently, pedagogical trends have promoted a more relaxed learning environment with an emphasis on making learning enjoyable [2]. Humour is a crucial behavioural trait and common in human communication [3] and is also among the main sources of happiness in the workplace [4]; it offers extensive advantages to employees and teams [4, 5]. Moreover, humour facilitates positive outcomes at work [4], increases positive evaluations of others, and deflects attention from negative information [6]. It draws attention and admiration, mitigates criticism (resulting in greater acceptance), sets social boundaries, and alleviates conflict between people with diverse viewpoints [7]. Furthermore, humour also helps people to handle anxiety, shyness, and sorrow [7]. The effective use of humour improves creativity [5, 8] enhances psychological resilience, alleviates stress, creates bonds, and improves status. At a group level, research shows that humour enhances team productivity and creates a positive atmosphere [2, 3, 9].

Universities are generally considered an appropriate context in which humour can be used as a communication tool [10, 11]. The study of humour in the context of educational leadership is a relatively new phenomenon [2]. In an educational atmosphere, a sense of humour induces feelings of conviviality and immediacy as opposed to feelings of isolation and alienation [10, 12]. Humour can improve cohesion and solidarity within a department [13, 14], and the chairs of educational departments (regarded as group leaders) can improve group intimacy through a sense of humour.

The chairs of educational departments are leaders who do not have the power or hierarchical tools to allocate economic resources and mostly play a facilitating role among the members of a group; therefore, they can use a sense of humour as a valuable socioemotional resource [4]. Educational departments are prone to factionalism and misconceptions about injustice and enjoyment, and chairs of departments are often required to manage disputes between younger and older members of a department in meetings. The chair of a department can diffuse such conflicts by speaking effectively and uttering a masterful sentence at the right time in proportion to the mood, atmosphere, and conditions. In all ongoing interactions of subtle forces, the chair of a department plays an important role [15]. A sense of humour is considered an appropriate way to seek a solution; however, a leader must know how to employ their sense of humour [16]. The chairs of educational departments can also use humour to alleviate stress and anxiety [3, 7]. If humour is used correctly in an acceptable and appropriate way, they can improve the effectiveness of educational departments, facilitate interactions [4], reduce the distance between themselves and staff, encourage professors to work harder, and enhance creativity and participation in group discussions by providing a comfortable atmosphere [2, 8, 13, 17]. Consequently, this process can enhance the effectiveness and performance of educational departments. A department chair’s sense of humour can probably promote the use of humour among professors in their interactions with students by creating an appropriate atmosphere [3, 9]. Studies have shown that a professor’s sense of humour in the classroom increases student satisfaction, enhances academic effort and participation [11], and improves students’ academic achievement [10].

Nevertheless, the topic of leader humour, especially in higher education, appears to be an area where scholarly inquiry has not kept pace with interest in the role of humour in leaders of organisations. Thus, a more comprehensive understanding of the subject should draw extensive practitioner appeal in addition to having theoretical value [4]. Scholarly interest in humour appears to be growing, as evidenced by the increasing number of publications on the topic over the last five years [18]. Existing studies on humour are often quantitative. In this regard, two meta-analyses by Mesmer-Magnus et al. [13] and Kong et al. [18] are among the more prominent. A few qualitative studies (e.g [19]). have tried to determine the outcomes of humour through interviews without taking a comprehensive look at the concept. Most researchers have based their studies on the content of humour, although the effects of humour and its success depend on a wide variety of factors in addition to the content of the message [20], something that has not received much emphasis. Studies have also neglected the positive nature of humour and the transcendence of sense of humour, nor have they distinguished between the two concepts. Many studies have paid little attention to the subtle differences between humour and sense of humour, and these two concepts have sometimes been mixed. A study that was published in top-tier journals used a humour questionnaire to measure sense of humour (e.g [21]). With a few exceptions (e.g [22]), no serious attempt has been made to identify the antecedents of humour in leaders. In the area of education, humour scholars have mainly focused on the use of humour in classrooms [2] and have neglected to address its effect on professional relationships in educational environments. Therefore, it is necessary to conduct a thorough qualitative study on sense of humour, with a focus on different aspects. Grounded theory (GT) appears to be an appropriate solution in such cases.

GT is used for various reasons such as the need for a broader theory or ambiguity in some aspects of a process ([23]; p. 450). Despite the significant growth in the number of studies on leader humour, a brief literature review indicates that the subject has not yet been analysed using GT. Although there are many empirical studies on the consequences of leader humour and the mechanisms for the conveyance of these outcomes, theoretical perceptions of these mechanisms remain limited and fragmentary [4]. Recent studies on leader humour have highlighted some ambiguities, problems, and contradictions regarding the conceptualisation and clarification of humour and sense of humour and warned about the necessity for major preliminary ‘housekeeping’ in this area [18]. Difficulties in research or theory construction are often caused by ambiguities and complexities in the underlying concepts [24]. The concept of leader humour is vague and requires a more accurate and comprehensive underlying conceptualisation. GT analysis can be a small step toward resolving ambiguities and complexities in this area.

## Method

GT is an inductive research method in which the theory emerges from the data and is grounded in it [25, 26] According to Glaser [25], a theory should emerge from the data, but researchers should not consider the relationships between the categories through axial coding in advance. The researcher should then seek the categories that are compatible with this pattern (i.e. the coding paradigm). In GT, this approach has no predefined assumptions but discovers the main concerns of the participants and how to resolve these concerns. This approach explains how participants deal with their major concerns, even though they might not be aware of the problem conceptually [25, 26].

We invited professors in educational departments at top Iranian universities to participate in this study. A theoretical sampling method was employed to select the research sample. According to Glaser [25], researchers should select individuals and sets and sample them purposively to collect useful information regarding their area of interest. In this study, theoretical sampling was performed until the categories were theoretically saturated [27] and no new data emerged regarding the categories. In qualitative studies, especially those based on GT, it is important to select individuals who can help researchers achieve their research goals [27]. In this study, efforts were made to select individuals with the most expertise, namely, professors with prior experience as department chairs. The participants were also expected to have experience in interacting with at least two different departments to meet the criteria for ‘having a key role’ and ‘reaching the theoretical perception of the matter’. The participants were completely familiar with the atmosphere and culture governing the designated universities, faculties, and educational departments; therefore, they could comment on the sense of humour of their institution’s leaders (chairs of departments). In every interview, the interviewee was asked to introduce teachers who had or welcomed a sense of humour so that the criterion for ‘being identified by other’ could be met. The participants were also selected from different faculties, departments, and genders so that the resultant data would have universality and comply with ‘diversity’ criteria.

This study utilised semi-structured and in-depth interviews to obtain the most appropriate data for a GT study [28]. Given the fundamental nature of qualitative research, the decision regarding the best data collection methods and from whom and how to collect data was finally made in the field of study and while conducting it. The interview usually began with open-ended and non-judgmental questions such as: ‘What is the right definition of humour in your opinion and what are some examples of it?’ and ‘Which strategies and methods does the chair of your department (leader) usually use to express their sense of humour? And which one has usually been successful and has received a positive response from the audience?’ Subsequent follow-up and probing questions were selected based on the participants’ initial responses and field notes to collect additional data on the topic under discussion. For example, the participant was asked to further explain or provide examples of humorous behaviours, causes, and outcomes. After each interview, the research team analysed the accumulated data, and the following interview was conducted based on the information obtained.

Each interview lasted approximately 60 min. According to prior coordination and permission from the participants, all interviews were recorded by a digital tape recorder and were then written and transcribed verbatim.

Based on Rubin and Rubin [29], who proposed interpretive analysis of texts in notes, the following steps were taken:

1. The interviews were first recorded.
2. The recorded interviews were then transcribed.
3. The main points were highlighted in the text, and repetitive words, statements, trivial items, and other irrelevant data were excluded.
4. Similar words that seemed to express new points were highlighted.
5. After all the texts were reviewed, the primary texts were reviewed to ensure that important points were not neglected while highlighting the main points. The second researcher was asked to mark the text separately. The individual marks were then compared and necessary changes were made.
6. The researcher started with the first text again, reviewed the highlighted points to extract a series of items from the responses given to the questions, and allocated simple titles to them. A large number of items were extracted from the first texts that were transcribed; however, the number of extracted titles decreased gradually in subsequent texts because the respondents mentioned similar points.
7. In this step, the list of items was analysed. Some of the items were combined, whereas some titles were deleted as appropriate or necessary. This process was repeated after the final analysis, and some names and classifications were removed.
8. After the final categorisation system was determined, the interviews were transcribed again, and every highlighted word was compared with the list of items. Question marks were placed in front of statements that could not easily be related to any item. In some cases, words were changed in the titles of items, or new items were added to match the sentences better and include sentences that were either questioned or not among the main responses.
9. The items were then inserted into the analysis table.

The first six interviews were conducted over short intervals. The data were then entered into the software after the interviews were analysed line by line several times. Other interviews were conducted through theoretical and cognitive sampling to develop the theory and enrich its sections.

Data analysis followed Glaser’s [25] approach, employing open coding, selective coding, and theoretical coding. MAXQDA2020 software facilitated data management and analysis. The 6 C family model [25] guided the organisation of identified codes into categories representing causes, consequences, contingencies, contexts, conditions, and covariance. Theoretical coding with the 6Cs framework facilitates a deeper understanding of data by prompting researchers to analyse relationships between categories. This method encourages asking questions about causality, intervening conditions, context, contingency, and covariance. Examining whether a category is a cause or consequence of another unveils potential causal links. Identifying intervening conditions provides insights into the mechanisms through which these relationships operate [30]. Contextualisation situates categories within specific events or incidents, revealing their relevance. Analysing contingency explores how changes in one category depend on others, highlighting potential dependencies and unplanned changes. Assessing covariance uncovers correlated changes between categories [31]. By systematically addressing these questions, researchers can progressively elevate the level of abstraction in their analysis, leading to a more comprehensive and nuanced understanding of the data. ‘Causes’ reflect questions aimed at considering the reason or explanation for the occurrence of a given phenomenon. ‘Consequences’ are the effects of the phenomenon. In terms of the nomothetic approaches to research, these two Cs reflect the relationship between dependent and independent variables. A ‘contingency’ is, in effect, a moderating variable. An intervening ‘condition’ is, in effect, an intervening variable. ‘Covariance’ between categories is equivalent to correlation. ‘Context’ accounts for the setting and events imposed on the setting [30]. The present research also pursued its objectives by going through these three main steps in such a way that in the open coding stage, free data coding continued until the emergence of the core category. When the core category was illustrated, selective coding began, and interviews were coded in the direction of the core variable. After selective coding, the extracted concepts were integrated to extract the model.

Based on the data obtained from the real coding, it became evident in the theoretical coding step that the 6 C family [25] provided the best possible combination of items within the axial category. It included a series of words related to causes, consequences, contingencies, contexts, conditions, and covariance.

In the open coding step, which is considered the first step in explaining the coding process, all the interviews were converted into codes on a sentence-by-sentence basis. For this purpose, important concepts were highlighted in the text and separate primary codes were determined. Consider the following segment:

*Of course*,* we need to know each other well enough so that we don’t get upset. It’s important to create a friendly atmosphere. I don’t want to just make people laugh. If it makes somebody upset*,* it’s not called humour anymore*,* although a few people laugh.*

The primary codes ‘knowing audiences’, ‘knowing the leader’, ‘conviviality’, and ‘invasive concepts’ were extracted from the above segment. Consider the following segment:

*Every action receives a reaction. If I kid someone who misunderstands the situation*,* I won’t definitely do it again*,* but if I do it and see the atmosphere changes*,* it might be positive. This depends on my perception of the environment.*

The primary codes ‘perceiving the time and place of humour’, ‘experience’, and ‘refining the environment’ were extracted. In this step, the axial item was identified as ‘humorous leader behaviour’. After the primary codes were identified, the second step involved the selective coding of the axial item. Approximately 180 primary codes were identified in the two steps. Subsequently, codes with similar concepts were combined to obtain 110 primary codes. Due to the large amount of content, we only present one instance (Table 1). This procedure was followed for all the primary codes, secondary codes, and items. The primary codes of similar and close concepts established 34 secondary codes. Similar secondary codes were then placed together to form levels with appropriate titles. Finally, 34 secondary codes were classified into 14 categories (Table 2).

**Table 1:** An instance of open coding, selective coding, and theoretical coding procedures (leader sense of humour)

Instances	Open coding and selective coding			Theoretical coding
	Primary codes	Secondary codes	Level	Category
* - Some people think that humour is not suitable for the academic environment and might say, ‘What is this nonsense?!’ This is where we must recognise the situation. The norms of a university differ from the norms of a private company! The opposite is also true, which means a good joke at a university may fall flat at a company.* - *The limits of humour mean that it should not upset anybody. People should not be offended, and jokes should not violate social norms.*	Knowing the norms in the context	Perceiving situations and conditions	Components of leader sense of humour	Cause
*- The situations in which humour is used can have an impact. For example, I’m dealing with a family problem today, so humour makes me upset because I’m not feeling well, so humour can be misunderstood.* *- It depends on experience, by which you can understand what joke suits what situation and for whom.*	Knowing situations based on experience			
*- Every action has a reaction. If I kid someone who misunderstands the situation, I definitely won’t do it again, but if I do it and see the atmosphere changes, it might be positive. This depends on my perception of the environment.* - *It depends on experience, by which you can understand what joke suits what situation and for whom.* *- A successful humorous person is someone who knows how to kid people properly at the right time. Such an individual should know the other party and realise what level of details should be used. This is what a joker should understand and be clever enough to know the contingencies and conditions.*	Understanding the right time and place for humour			
*- These people (humorous people) are the ones who, when they become aware of the problems of working with others, usually come up with a solution easily in their mind, through a trick and a joke.*				
*- For example, in the recent department meeting there was a serious discussion about theses and a confrontation was about to happen. One of the younger people said I have been at the university for 7 years and have seen many defenses, and it should happen this way. In response, another person said I have been at the university for 29 years and didn’t intend to confront or didn’t mean anything. I said that a confrontation might happen and quickly made it humorous and said that you equated those 7 years of the poor guy to dust; everyone laughed and the atmosphere changed.*	Finding solutions in communication	Creativity		
*- For example, we had a meeting with PhD students where they had brought a gift for one of the students. One of the students said what a bad colour these bags have and what did you do that they brought this for you, and the person said I didn’t give anyone a grade to do this and I don’t even know who it is for. I saw that the remark was heavy and jokingly said if you don’t know whose it is, this center is in my name so they must have brought it for me, and everyone laughed and the discussion ended; the start of an argument and formation of a misunderstanding can be changed with a joke. In some cases, the atmosphere is so heavy that this joke has to be used several times to fix the atmosphere.*				
*- Since it is spontaneous, it is related to creativity.*				
*- The person (department chair) should be innovative, not imitative. If he is imitative, he may be able to bring some topical entertainment in some cases, but in many cases, he is doomed to failure. Sometimes, people who have humorous traits show their distinction from others more with the humour.*	Understanding the humorous aspects of subjects			
*- Sense of humour cannot be planned. Humorous people usually don’t think and joke spontaneously.*				

**Fig. 1:**
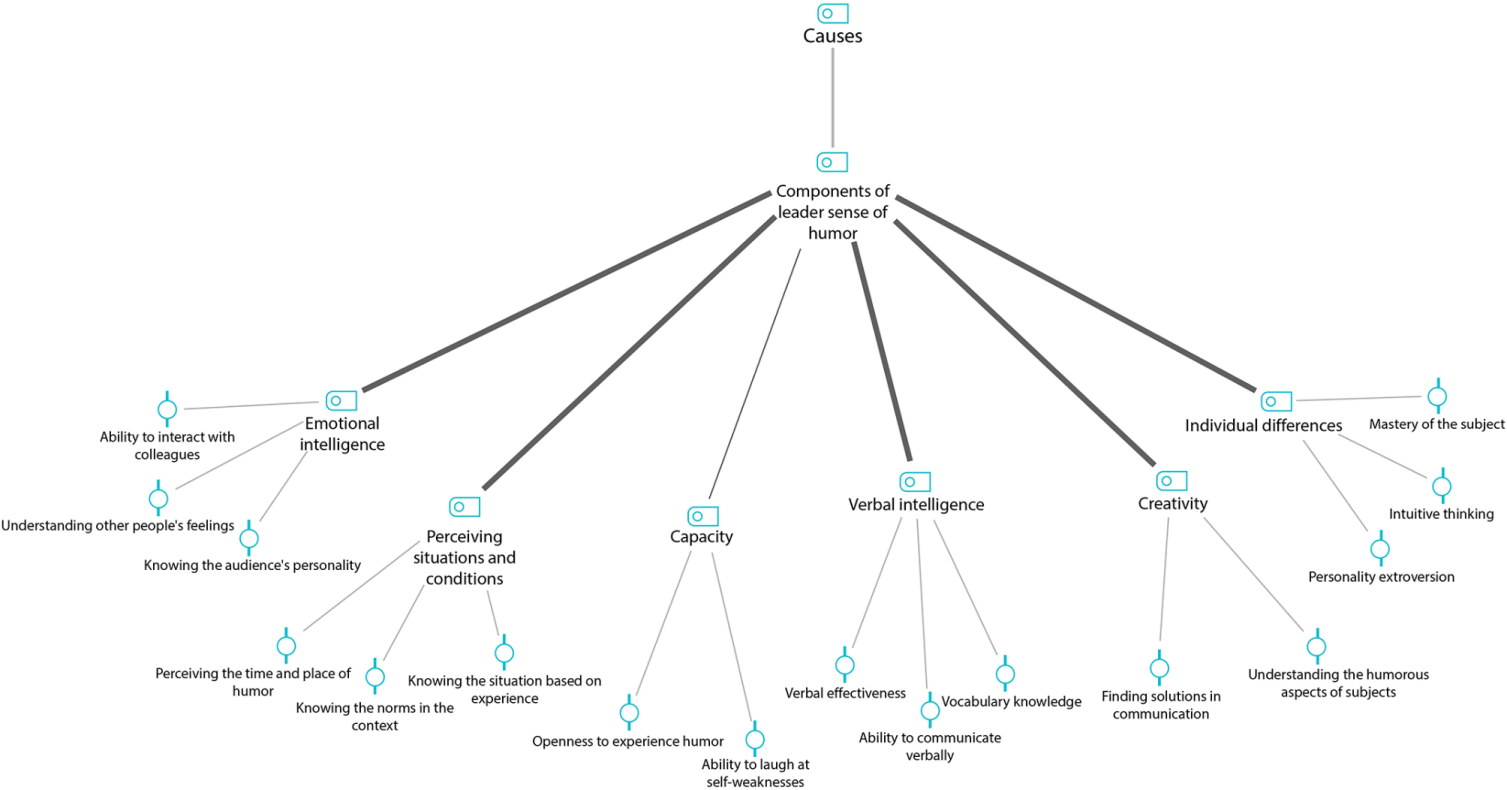
MAXQDA graph for causes

**Fig. 2:**
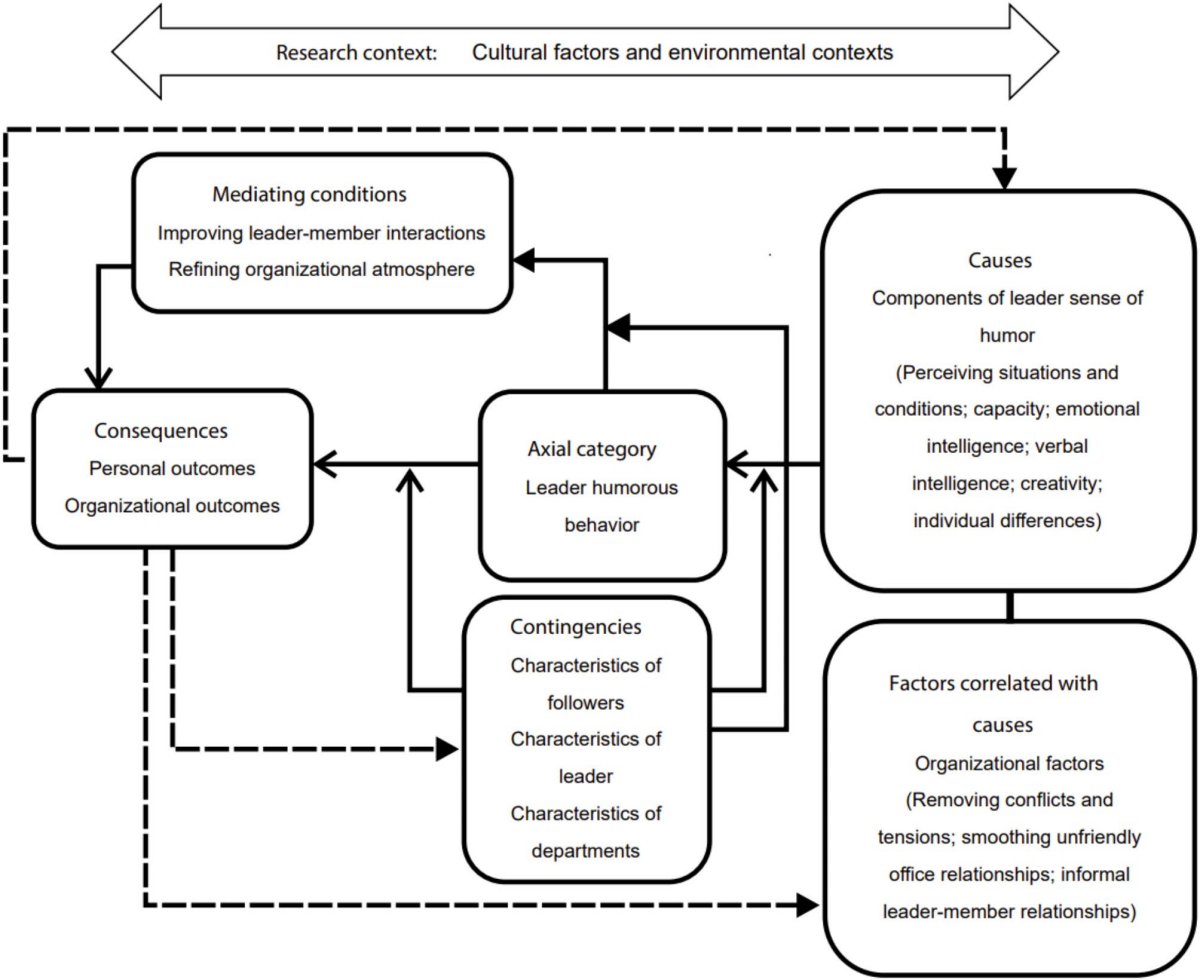
Exploratory research model

Similarly, the primary codes ‘the ability to laugh at self-weaknesses’ and ‘not being upset at jokes’ were extracted from the instances. They formed the secondary code ‘capacity’, whereas the primary codes ‘knowing the audience’s personality’, ‘perceiving other people’s feelings’, and ‘the ability to interact with others’ formed the secondary code ‘emotional intelligence’. The primary codes ‘the ability to verbally communicate with others’, ‘vocabulary knowledge’, and ‘verbal effectiveness’ produced the secondary code ‘verbal intelligence’. The primary codes ‘finding a solution to communications’ and ‘perceiving the humorous aspects of subjects’ resulted in the secondary code ‘creativity’, whereas the primary codes ‘personality extroversion’, ‘intuitive thinking’, and ‘mastery of the subject’ produced the secondary code ‘individual differences’. The secondary codes ‘capacity’, ‘perceiving situations and conditions’, ‘emotional intelligence’, ‘verbal intelligence’, ‘creativity’, and ‘individual differences’ formed the level ‘components of leader sense of humour’ that was considered the ‘cause’ of humorous behaviour. Figure 1 shows the MAXQDA graph for causes, Table 2 presents the codes and levels obtained from the data analysis, and Fig. 2 shows the final model.

**Table 2:** Codes and levels resulting from data analysis in selective coding

No.	Primary codes	Secondary codes	Levels
1	Ability to laugh at self-weaknesses	Capacity	Components of leader sense of humour
2	Openness to experience humour		
3	Knowing the norms in the context	Perceiving situations and conditions	
4	Knowing the situation based on experience		
5	Perceiving the time and place of humour		
6	Knowing the audience’s personality	Emotional intelligence	
7	Understanding other people’s feelings		
8	Ability to interact with colleagues		
9	Ability to communicate verbally	Verbal intelligence	
10	Vocabulary knowledge		
11	Verbal effectiveness		
12	Finding solutions in communication	Creativity	
13	Understanding the humorous aspects of subjects		
14	Personality extroversion	Individual differences	
15	Intuitive thinking		
16	Mastery of the subject		
17	Preventing and diffusing tension	Removing conflicts and tensions	Organisational factors
18	Preventing confrontation and disputes		
19	Humour in difficulties		
20	Reducing work stress and pressures	Smoothing office relationships	
21	Improving emotionless office relationships		
22	Breaking an unfriendly atmosphere		
23	Decreasing formality		
24	Cheerfulness in relationships	Informal relationships between leader and members	
25	Conviviality between leader and follower		
26	Sympathy		
27	Introducing a feeling of engagement to the department		
28	Humour at the right time	Improvisation	Humour expression
29	Humour in the right situation		
30	Brief words	Wit	
31	Surprising the audience with specific words		
32	Complicated expressions		
33	Proverbs	Prefabricated patterns	
34	Tales		
35	Poems		
36	Social media content		
37	Hypothesis		
38	Knowledge of current topics of interest		
39	Native accent		
40	Benevolent intention	Benign violation	Attributes of humour
41	Normative acceptability		
42	Incongruity		
43	Avoiding extremism	Moderation	
44	Avoiding offensive concepts		
45	Avoiding farce		
46	Humorous responses	Verbal	Reaction to humour
47	Body language	Nonverbal	
48	Increased popularity of leader	Leader-related outcomes	Individual outcomes
49	Increased legitimacy of leader		
50	Creating charisma for the leader		
51	Improving personal performance	Follower-related outcomes	
52	Improving job satisfaction		
53	Improving creativity		
54	Improving motivation		
55	Improving commitment		
56	Increasing the intention to stay		
57	Job embeddedness		
58	Showing extra-role behaviour		
59	Increasing intimacy between members	Organisational micro-outcomes	Organisational outcomes
60	Deepening relationships		
61	Sharing knowledge		
62	Increasing solidarity and cohesion		
63	Progressing goals	Organisational macro-outcomes	
64	Improving organisational performance		
65	Organisational support		
66	Reducing the power distance		
67	Followers’ literacy	Demographics of followers	Characteristics of followers
68	Followers’ career		
69	Gender similarity between follower and leader		
70	Follower sense of humour	Behavioural and personal characteristics of followers	
71	Previous experience		
72	Follower insight into the leader		
73	Cynicism		
74	Follower humility		
75	Sensitive personality		
76	Prejudice		
77	Malice		
78	Leaders’ literacy	Demographics of leader	Characteristics of leader
79	Leaders’ career		
80	Age similarity between leaders and followers		
81	Justice	Behavioural and personal characteristics of leader	
82	Charisma		
83	Lack of explicitness		
84	Lack of hypocrisy		
85	Leaders’ humility		
86	Department size	Demographics of a department	Characteristics of a department
87	Department composition		
88	Department cohesion	Behavioural characteristics of a department	
89	Department norms		
90	Department acceptance of an individual		
91	Leader effectiveness	Improving leaders’ position	Leader-member exchange improvement
92	Trust in leader		
93	Increasing energy	Stimulating positive emotions of followers	
94	Psychological well-being		
95	Improving human relationships	Improving organisational communications	Refinement of the organisational atmosphere
96	Reducing social distance		
97	Creating happiness	Positive environmental atmosphere	
98	Increasing cheerfulness		
99	Improving joyfulness		
100	Respect culture	Cultural empowers	Cultural factors
101	Low power distance		
102	Similarity between ethical and local culture		
103	Department, faculty, and university culture		
104	Joint research projects	Research and scientific collaborations	Environmental contexts
105	Joint theses and dissertations		
106	Joint scientific papers		
107	Different groups of professors on social media	Social media	
108	Direct two-sided relationships on social media		
109	Formal meetings of departments	Meetings of educational departments	
110	Informal meetings of departments		

## Results and discussion

As discussed earlier, the elements of the research model were classified into six categories and one axial category in the 6 C family model. The axial (core) category functions as the core concept that integrates all the other categories identified in the analysis. It represents the central phenomenon around which the causes, consequences, contingencies, contexts, conditions, and covariance are organised and explains how participants continuously resolve their main concerns. The axial category emerges through the coding process and helps to explain the interrelationships among the various categories [25, 28]. In this study specifically, the axial category was identified as ‘leader humorous behaviour’ through the theoretical coding process. This central category ties together the other elements of the theoretical model (6Cs). The first ‘C’ stands for ‘Causes’, which reflects the reasons and explanations for the occurrence of the axial category [30]. In this study, ‘leader sense of humour’ emerged as the cause of the axial category, ‘leader humorous behaviour’. Data analysis revealed that individuals fundamentally require a sense of humour to exhibit successful humorous behaviour. The elements of sense of humour include capacity, the ability to perceive situations and conditions, possessing verbal and emotional intelligence, creativity, and certain individual differences. The second ‘C’ represents ‘Covariance’, referring to factors correlated with the causes of the main process in GT, changing in tandem with them [30]. This study identifies organisational factors as covarying with the axial category, as leader humorous behaviour cannot manifest in the workplace without certain organisational factors. Specifically, merely possessing the elements of sense of humour is insufficient; the expression of humorous behaviour in the workplace necessitates certain conditions, motivations, and relationships; including removing conflicts and tensions, smoothing office relationships, informal relationships between leader and members. The third ‘C’ stands for ‘Contingencies’, referring to moderating factors that influence the relationships between categories [30]. In this study, the identified contingencies are factors that moderate the relationship between the axial category and its causes, as well as the relationship between the axial category and its outcomes and mediating conditions. These factors include the demographic and behavioural characteristics of the leader, followers, and the group. The fourth ‘C’ represents ‘Conditions’, referring to mediating variables [30]. In this study, conditions, or mediating variables, are those that emerge and take shape before the appearance of the final outcomes resulting from the axial category. The mediating variables identified in this study are improved leader-member exchange and a more refined organisational atmosphere. The fifth ‘C’ stands for ‘Consequences’, referring to the outcomes resulting from the axial category [30]. In this study, the axial category has multiple consequences, broadly categorised as individual and organisational outcomes. The sixth ‘C’ represents ‘Context’, referring to the setting or environment in which the research is conducted [30]. The environmental context of this study reveals two main characteristics: cultural factors (cultural empowers) and environmental settings (including research and scientific collaborations, meetings of educational departments, and social media).

### Leader humorous behaviour

In this study, the axial category serves as the central phenomenon around which all other categories, such as the components of leader sense of humour, organisational factors, mediating conditions, contextual factors, and outcomes are integrated. The axial category named ‘leader humorous behaviour’ was identified through the coding process and represents the core concept that explains the relationships between the other categories in the theoretical model. ‘Leader humorous behaviour’ is considered the behavioural outcome of ‘leader sense of humour’. The term ‘humorous behaviour’ was used instead of ‘humour’ because sense of humour sometimes manifests itself as a reaction to other people’s humour in addition to self-humour expression. This important distinction differentiates ‘humorous behaviour’ from ‘humour’. Humorous behaviour includes the humour expression and reactions to humour (verbal or through body language).

In this study, humour expression refers to the early expression of humour by a leader concerning the predetermined and conventional patterns of humour, improvisation, and wit that occur spontaneously in appropriate situations and contexts. Reaction to humour refers to how a leader reacts to other people’s humour and includes both verbal and nonverbal responses. Sometimes, a leader does not initiate humour; however, their response to other people’s humour reveals their sense of humour. This response may be verbal (e.g., responding with a humorous statement) or nonverbal (e.g., a timely smile). In both instances, the response indicates the leaders’ sense and perception of humour.

Consistent with the results of this study, Martin and Ford [2] implicitly pointed out the responses and reactions to other people’s humour. Holmes [32] stated that humour usually requires a response. Adopting a linguistic approach, Schnurr and Chan [33] focused on followers’ responses to leader humour and identified the factors that affect follower responses to leader humour, such as perceptions of humour, norms, and cultural factors. Although Schnurr and Chan [33] did not regard responses to humour directly as humorous behaviour, they provided concepts and explanations for responses to humour to indicate that it would be included in humorous behaviour. In this study, humour expression includes improvisation, witnessing, and the use of predetermined patterns. Improvisation results in spontaneous humour expression and the use of humorous words based on circumstances is consistent with the results reported by Gervais and Wilson [7].

Moreover, ‘wit’ refers to unexpected, short, pithy, and funny expressions. Since the results of this study emphasise benevolent and positive intentions in humour, we strongly disagree with some of the classifications of Long and Graesser [34] and Koestler [35], who introduced humorous actions as unintentional and regarded sarcasm as humour. Instead, this study focuses on the predetermined patterns used by leaders to express humour, such as proverbs, tales, poems, social media content, hypotheses, knowledge on topics of current interest, and use of native accents. Some of these classifications emerge from Iran’s cultural background.

Based on the analysis of the interviews, the main attributes of humour—in addition to being funny and provoking laughter—were identified as (i) benign violation and (ii) moderation. In benign violation, the emphasis is on humour with benevolent intentions, normative acceptance, and incongruity in the components of humour (quality of humour). In moderation, the emphasis is on the importance of avoiding extremism, offensive concepts, and farce (frequency of humour expression). Specifically, an expression of humour could be interpreted as a farce if it is accompanied by concepts that are considered demeaning. An expression of humour could be interpreted as offensive if it is accompanied by humiliation, sarcasm, or mockery, which would qualitatively be considered beyond the limits of humour. In addition, when leader humorous behaviour shows no sign of demeaning concepts, mockery, or humiliation of followers but is repeated too often, it will quantitatively be considered beyond the boundaries of humour.

In this study, the resultant attributes of humour include benign violations, consistent with the studies of McGraw and Warren [36] and Warren and McGraw [37], as well as the incongruity theory [38]. According to the incongruity theory, humour arises from the perception of incongruity between a concept and the real objects thought to be in some relation to it [38]. Similar to the views of Martin [39], Ruch [40], Cooper et al. [4], and Hobfoll [41], this study generally considers sense of humour a positive and transcendental personality trait that presents at different levels. This study argues that a high level of sense of humour helps to correctly perceive the conditions, norms, feelings, contingencies, and humorous aspects of subjects and prevents leaders from using offensive concepts or applying force in addition to perceiving the appropriate number of repetitions (and avoiding extremism) to express humorous behaviour. Specifically, sense of humour is inherently a transcendental construct; without any limitations, it is never considered excessive or too much. However, humour as a behaviour requires moderation (and avoidance of extremism) because such behaviour might originate from a low level of sense of humour, in which case, repeating humorous behaviour that does not even represent farce and offensive concepts will end up offending the audience.

To our knowledge, this study appears to be among the first to highlight the importance of moderation in humorous behaviour (in terms of the frequency and repetition of humorous behaviour) as a significant attribute of leader humour alongside benign violation. Earlier studies reported a few negative consequences of humour based on its content (e.g [9, 21, 42]), but did not investigate the probability of negative consequences of completely positive humour (having benevolent intention). According to the results of this study, even leader humour with completely positive and constructive content could have negative and unwanted outcomes for both the leader and their followers in the case of extremism (lack of moderation). As a result, humour could be interpreted as mockery and reticulation. The necessity of considering extremism in humour can be interpreted through too-much-of-a-good-thing (TMGT) meta-theory [43].Accordingly, even if humour is expressed through completely positive content, it can quantitatively violate the boundaries of ‘being humorous’ and be considered instances of farce and offensive behaviour in the case of too many repetitions. Deviating from the boundaries of being humorous is also justified qualitatively (as offensive concepts and farce) due to benign violation theory [36], because using offensive concepts will make the violation exceed the boundaries of ‘being benign’. Farce is an expression of benign behaviour; however, it is not considered a ‘violation’; therefore, it fails to generate laughter and joy.

### Components of leader sense of humour

Causes reflect the reasons and explanations for the occurrence of the axial category. In this study, the first identified item, that is, the causes of humour that reflect the axial category, was named ‘components of leader sense of humour’, which is the hidden and unexpressed aspect of their humorous behaviour. Analysis of the interviews indicates that people usually need a sense of humour to successfully express humorous behaviour. The data suggest that components of sense of humour include capacity, the ability to perceive a situation, verbal intelligence, emotional intelligence, creativity, and individual differences. It is basically due to the above characteristics that people engage in humorous behaviour. As discussed earlier, many studies disregarded the difference between sense of humour, humour, and humorous behaviour (e.g [21]. ). According to the results of this study, ‘sense of humour’ refers to a series of completely positive personal attributes and characteristics that cause humorous behaviour (including humour). Specifically, any kind of humorous expression, perception of humour, or reaction to humour is rooted in a person’s sense of humour. Earlier studies have addressed the cause-and-effect relationship between sense of humour and humour (as an instance of humorous behaviour) (e.g [2, 4, 13]. ). However, the results of this study are inconsistent with some of the abovementioned studies (e.g [13]. ) that considered humour the only emerging aspect of sense of humour. We argue that humour is an instance of sense of humour expression, which itself includes any perception of humour such as reactions to humour. Moreover, sense of humour manifests as humorous behaviour, and an instance of humorous behaviour (which is the most evident one) as humour.

In this study, capacity refers to the ability to laugh at one’s weaknesses and openness to experiencing humour without becoming upset at other people’s mistakes. Some studies referred to capacity as ‘not taking oneself seriously’ and an ‘ability to laugh at self-weaknesses’ [44, 45]. This study also confirms the relationship between intelligence and sense of humour [2, 46, 47]. Leaders with high levels of verbal and emotional intelligence and creativity can better perceive and express the humorous aspects of their subjects. In addition, higher levels of emotional intelligence help individuals to perceive humorous conditions. Although a sense of humour is associated with intelligence and creativity in leadership studies [2, 47], creativity (of followers, not the leader) is generally considered an outcome of leader humour (e.g [5, 8, 14]. In this section, creativity refers to a leader’s ability to exhibit humorous behaviour based on circumstances, whereas followers’ creativity is introduced as an outcome of the humorous behaviour expressed by the leader.

Simultaneously, personality extroversion, intuitive thinking, and higher mastery of a subject of interest can help leaders use their sense of humor in a given situation rather than suppressing it. For example, the leader’s extroversion makes him expose himself more to humorous situations. Additionally, the more the leader has mastery over the subject under discussion, the more he sees his hand free in using his sense of humour and consequently presents appropriate humorous behaviour. Martin et al. [48] confirmed the effect of personality extroversion on the expression of positive humour; however, this study introduces intuitive thinking and mastery of a subject as components of a leader’s sense of humour. Perception of a situation is another component of sense of humour. The perception of situations and conditions refers to the accurate identification of the right time and place for an acceptable and appropriate expression of humour. Accordingly, a humorous person is one who identifies the right context and atmosphere to express appropriate humour.

Consistent with the results of this study, most researchers agree that sense of humour is a personality trait that allows a person to recognise and use humour appropriately [13, 47, 49, 50]. Among the four dimensions of sense of humour, Thorson and Powell [50] introduced the second and third dimensions in their multidimensional sense of humour scale (MSHS) to recognise and appreciate humour as well as humorous people and situations. In the sense of humour questionnaire, Svebak [51] pointed out dimensions such as the ability to notice humorous stimuli in one’s environment, in addition to the ability to express and suppress humorous emotions. Researchers generally agree that sense of humour, regardless of style, is a stable personality trait that creates a propensity to use and recognise successful humour (e.g [2, 13, 40]). Kong et al. [18] posed a provocative question: is leader trait humour (sense of humour) as an individual characteristic needed by itself concerning the weaker value in its association with all follower outcomes in comparison with leader humour expression (proven in their meta-analysis)? In response to this question, they argued that a leader’s sense of humour is a latent quality and can be effective only when it is manifested as humour.

The results of this study add to the conclusions of Kong et al. [18] by stating that although the components of leaders’ sense of humour are latent, they are significantly more important and valuable than expressions of humour. Higher levels of sense of humour result in the perception of situations, conditions, and emotions, which leads to the successful expression of humour and prevents extremism or violations of humour limits. Furthermore, leaders’ sense of humour could manifest as an effective reaction to other people’s humour rather than an expression of humour. Their high perception of situations can thereby prevent tension, conflict, and inappropriate behavioural outcomes in both leaders and their followers. Hence, a leader’s sense of humour should be considered significantly more valuable than leader humour expression.

### Correlated causes

Correlated causes are factors associated with the primary causes in grounded theory and vary along with these primary causes. In this study, organisational factors are identified as correlated with the axial category. Particularly, as organisational factors increase alongside humorous individuals, the frequency of humorous behaviour also increases. Based on data analysis, the second identified item is correlated causes, which include organisational factors. Converting the components of sense of humour into behaviour, organisationally correlated causes pertain to the conditions and motivations that stimulate and encourage leaders’ expression of sense of humour, since leaders rarely express humorous behaviour at work unless some specific organisational factors are present. Specifically, the mere presence of the components of sense of humour in the leader is insufficient. The emergence of sense of humour at work requires specific conditions, motivations, and relationships. In this study, organisational correlated causes include motivation for resolving conflict and tension, smoothing office relationships, and informal leader–member relationships. Notably, the arousal [52] and relief theories of humour [2] have no relation to the aforementioned factors because according to these theories (e.g., superiority theory), humour is employed to relieve a person’s intrinsic stress and that their motivation for humour expression is to release their intrinsic stress rather than extrinsic tensions in the surrounding environment. Unlike current humour approaches, relief theories adopt a more negative approach and often include potentially offensive concepts such as sarcasm. This contrasts with the approach taken in the current study.

According to the results of this study, leaders who seek to reduce conflict and tension in their departments tend to take crucial actions when such tensions arise. If they possess an appropriate sense of humour, they can diffuse a situation by expressing appropriate humour. Such disputes often occur in formal meetings owing to disagreements. In this study, conflicts and tensions refer to confrontations that typically occur in the organisation during discussions and group work. Preventing increased tension, confrontation, and arguments are among the reasons that make humorous people display humorous behaviour in group and organisational gatherings. As mentioned, humour is a purposeful behaviour with benevolent intention, and the motivation to resolve conflicts and tensions indicates the existence of this intention in the leader and stimulates his sense of humour. Emotionless office relationships refer to inflexible and strict relationships that usually arise in stressful conditions and work pressure and are seen in some government organisations. Emotionless office relationships are one of the factors that make people use humorous behaviour in their work and group gatherings to make these relationships flexible. Some studies have analysed the role of humour in resolving conflict and facilitating relationships; the role of sense of humour was also confirmed in reducing conflict and tension between people with different opinions [2]. In another study, Robert and Wall [53] introduced humour as a driver of positive emotions and a facilitator of relationships between friends. However, this function of humour is something that humans inherently understand; therefore, people use their sense of humour in times of conflict or in challenging relationships that they are motivated to resolve.

In this study, informal relationships between the leader and members encompass the joy in these relationships, the leader’s empathy and intimacy with followers, and the followers’ sense of belonging to the group. While softening rigid relationships and resolving tensions and conflicts provide the necessary motivation for the leader to express their sense of humour, the formation of humour requires a relatively joyful and intimate atmosphere. It is apparent that in a closed and unfriendly atmosphere, it is not possible to express a sense of humour, but the minimum environmental conditions are necessary for leaders to express humorous behaviour. Informal leader–follower relationships are also drivers of leaders’ humorous behaviour. Evidently, humour occurs in informal contexts, and leaders who feel more comfortable in their relationships with their followers will be less worried about followers misunderstanding their mistakes and will probably use their sense of humour more often. The results reported by Yang et al. [54] are consistent with those of this study, namely, that both leaders and followers stop being serious in an informal atmosphere and show more humorous behaviour.

### Factors related to context

The third identified category includes the factors related to context, which refers to the macro-level factors affecting leaders’ humorous behaviour, in addition to including the context for the formation of the axial item. This category includes cultural factors and environmental contexts. The results of the data analysis show that the respect culture, low power distance, and similarity between ethical, local, and departmental cultures facilitate the expression of sense of humour. Specifically, a respectful and value-driven environment enables individuals to express humorous behaviour effectively. A review of the literature on the relationship between sense of humour and culture shows an undeniable correlation between different levels of culture and humour. Different cultures have specific rules concerning humour and situations where laughter is appropriate, and cultural differences can affect the use of humour and the proper role of laughter [2]. Humour norms differ in industries, companies, and across cultures. Content that is fun in one culture may be perceived as confusing or even offensive in another [18]. Culture affects humorous behaviour at a national, ethical, organisational, and group levels. Considering that most studies on sense of humour are quantitative, they often overlook cultural factors at the macro level.

Many studies have considered the prominent role of culture in humorous behaviour. In this study, culture refers to national culture (e.g. the culture of common respect in Iran), ethnic culture (similarity between the local and ethnic cultures of members), and organisational culture (at the levels of departments, faculties, and universities). Many studies have focused on organisational culture. Most researchers considered organisational culture as a series of values, behavioural rules, customs, and common narratives that connect the members of an organisation and give them an identity [2]. Organisational culture emerges among individuals who cooperate, whereas humour appears to be a pervasive characteristic of these interactions at work [54]. A two-sided relationship exists between sense of humour and organisational culture. Scholars have indicated that, as organisational culture affects humorous behaviour in an organisation, humour also plays an important role in forming organisational culture [14].

Another two-sided relationship that emerged in this study is low power distance, which provides an appropriate context for the expression of humorous behaviour and is also a positive outcome of leaders’ humorous behaviour. The effect of humour on organisational culture is usually perceived through ‘humour climate’ [55, 56]. Blanchard et al. [56] defined humour climate as the shared perception of employees in a workgroup on how to use and express humour. In every organisation, a humour climate might be either positive or negative and can be beneficial or detrimental to the organisational culture by affecting the psychological well-being and individual performance of employees and the quality of their interactions [2]. Simultaneously, organisational culture may encourage leaders to engage in humorous behaviour or prevent them from exhibiting such behaviour [19]. Southwest Airlines is a prominent example. Moran and Roth [57] showed that an organisational culture based on humour is necessary to reduce tensions and create bonds between members. Dhillon et al. [58] addressed the role of humour in organisational culture when a company’s ownership changes and introduced humour as an appropriate solution for the resolution of cultural conflict. Person et al. [59] indicated that the presence of humour in the organisational culture of an emergency ward improved performance, despite staff members’ heavy workload. Universities and higher education systems are among the areas in which organisational culture plays a prominent role, and humour in the organisational culture of these institutions can affect students and education staff [19].

Regarding cultural factors, the results of this study indicate that culture affects the quality, quantity, and ways of expressing, interpreting, and perceiving sense of humour, humorous behaviour, outcomes, and contingencies at different national, organisational (university and faculty), and departmental levels. Moreover, similarities between the cultural and ethnic cultures of leaders and followers encourage them to express humorous behaviour and minimise misunderstandings. Another study demonstrated the necessity of paying attention to the formal and informal contexts of leader humour expression in organisations by analysing this behaviour in different contexts and countries [54]. Robert et al. [60] indicated that the context of humour is one of the factors that affect followers’ perceptions of leader humour. Reversal theory [61] focuses on the context in which humour manifests [2].

In this study, environmental contexts include formal and informal meetings of departments, contexts of research and scientific collaborations, and leader–follower communication in social networks. In a statistical population, humorous behaviour usually emerges in one of these contexts. Social media provide the widest context for the exchange of humorous content.

### Mediating conditions and consequences of leaders’ humorous behaviour

The fourth and fifth identified categories refer to the mediating conditions and consequences of leader humorous behaviour. The axial category has various results, including individual and organisational consequences and mediating conditions or variables are factors that shape the outcomes resulting from the axial category of the research before the outcomes appear. Specifically, mediating conditions are the initial outcomes through which the outcomes are shaped. The mediating conditions obtained in this research are generally divided into two categories: improving the leader-member exchange and refining the organisational atmosphere. Based on the obtained results, among the mediating conditions affecting the outcomes of the leader’s humorous behaviour are improving the leader’s position and stimulating the positive emotions of the followers, and among the mediating conditions affecting the outcomes of the leader’s humorous behaviour is refining the organisational atmosphere, which includes improving organisational communications and positive environmental atmosphere. Individual outcomes include the outcomes that arise in individuals as a result of expressing humorous behaviour. These outcomes may be specific to the leader or related to the followers. For example, improving individual performance, job satisfaction, increasing the desire to stay, and job attachment are positive outcomes of the leader’s humorous behaviours in followers. However, some individual outcomes of the leader’s humorous behaviours are specific to him; for example, the expression of humorous behaviours by the leader increases his popularity. Organisational outcomes are also the micro and macro outcomes that the leader’s humorous behaviour brings to the organisation. Leader-related outcomes include increasing popularity, creating charisma, and increasing legitimacy for the leader. The leader’s humorous behaviour has these positive results for him. Follower-related outcomes include attitudinal outcomes (job satisfaction, commitment, and job embeddedness) and behavioural outcomes (individual performance, creativity, motivation, intention to stay, and extra-role behaviours). The aforementioned cases are the outcomes of the leader’s humorous behaviour that appears in the followers. Organisational outcomes are the micro and macro outcomes that the leader’s humorous behaviour brings to the organisation. In this study, organisational micro outcomes refer to deepening relationships, improving intimacy between members, sharing knowledge, and group cohesion and solidarity. The meaning of group cohesion and solidarity in this research is the amount of effective communication between group members. Organisational macro outcomes include progressing goals, improving organisational performance, organisational support, and reducing power distance, which is among the benefits of using sense of humour by leaders at the organisational level.

In this study, the resulting outcomes and conditions resemble the mediating outcomes and factors determined by other scholars. Therefore, a similar study was conducted by Kong et al. [18], who introduced mediating factors such as leader–member exchange, followers’ trust in leaders, and followers’ positive emotions. Their meta-analysis also explained job performance improvement, organisational citizenship behaviour, follower job satisfaction, affective organisational commitment, and intention to stay in an organisation as positive outcomes of humour. The similarity between their study and this study lies in the mediating factors (leader–follower exchange, trust in leaders, and positive emotions of followers), as well as certain outcomes such as job performance, commitment, citizenship behaviour, and job satisfaction. The difference between the two studies lies in ignoring the role of a conducive organisational atmosphere in leader humorous behaviour outcomes and the lack of emphasis on the outcomes of leader humour for themselves (something which was analysed in this study).

Other scholars have independently confirmed the mediating role of leader–follower exchange in the positive outcomes of leader humour [21, 62], the effect of humour on the quality of leader–member exchange [62, 63], and the effect of leader humour on employees’ organisational citizenship behaviours [64]. In another meta-analysis, Mesmer-Magnus et al. [13] indicated that employees’ sense of humour improves job performance, job satisfaction, group cohesion, and physical health; and mitigates occupational fatigue and stress. A supervisor’s sense of humour can also improve followers’ performance, their (job) satisfaction, satisfaction with the supervisor, and group cohesion, as well as create a positive perception of the supervisor’s performance, which would also decrease occupational fatigue.

Although Mesmer-Magnus et al. [13] confirmed the difference between humour and sense of humour, they considered humour and sense of humour to be equal and two different aspects of the same phenomenon. Consistent with this study, other studies also identified and confirmed creativity [5, 8], followers’ job engagement [21, 60], increase in leader status [3, 9], improvement in followers’ psychological well-being [41, 63], greater group cohesion [14, 45], and improvement in levels of happiness, cheerfulness, and joy at work [65] as the positive outcomes of leader humour.

Consistent with previous studies, the mediating conditions and positive outcomes can be interpreted through the social exchange theory [41, 66], conservation of resources theory [42], broaden-and-build theory [67], and leader–member exchange theory [18, 68] in this study, because humour is considered a positive phenomenon and a valuable source and driver of positive emotions (for followers). Therefore, leaders can use humorous behaviours to improve the quality of their relationships with their followers, which could lead to positive outcomes.

Some researchers identified the negative outcomes of leader humour as deviant behaviour in followers [21] and decreasing the leader’s status [3, 9]. The important characteristic of this group of scholars is that they analyse offensive concepts, such as ‘sarcasm’, ‘mockery’, ‘ridicule’, and ‘banter’, in practice rather than the analysis of ‘humour’ (e.g [43]).; they also consider humour as any funny action in general [2, 21]. Thus, they based their studies on the positive and negative content of humour, and confused humour with similar and sometimes contradictory concepts. Therefore, these findings are inconsistent with the results of this study, which considers humour to have a real and positive meaning. Some determined outcomes (e.g. improving conviviality, increasing group cohesion, and increasing creativity) are similar and sometimes equal to the causes, correlated causes, and moderating causes in this study, indicating the existence of a recursive two-sided relationship between sense of humour and some of its outcomes. Specifically, the positive outcomes of leaders’ humorous behaviour increase humour expression in the organisation over time and prevent misunderstandings. In addition, they enrich a leader’s sense of humour by increasing their experience and helping them to better understand norms and situations.

Accordingly, it can be stated that the proposed model is dynamic because it yields unstopped outcomes and returns to other research categories (e.g. correlated causes, contingencies, and causes). Hence, it is dynamic rather than static in terms of rotation. This dimension of this study is consistent with the ‘wheel model of humour’ developed by Robert and Wilbanks [69]. According to this model, acceptable humour expression generates positive emotions, allows emotions to emerge, and has a positive effect on a department (or two-sided relationships between leaders and their followers). This provides the necessary supportive atmosphere for future expressions of humour. The cycle of this model exists in the relationships between leaders and followers through leader–follower exchanges [69]. However, the wheel model only emphasises the role of emotions in creating a supportive atmosphere for humour regeneration. According to the results of this study, the positive outcomes of humour increase the supportive atmosphere through positive emotions and leader–member exchanges in addition to refining the environment and improving conviviality by increasing leadership effectiveness, increasing experience, and improving creativity. At the same time, the successful expression of humour by a leader and the acceptance of followers will gradually allow them to get to know each other better. Through the accumulation of experiences on both sides, the chances of misunderstandings are minimised. If the misunderstanding factors decrease, the leader is motivated to exhibit further humorous behaviour. Therefore, the model proposed by Robert and Wilbanks [69] should also include the fact that the organisational humour cycle is not merely limited and bound to increase positive emotions but can also flow through other ways.

### Moderating factors; demographic and behavioural characteristics

The sixth category includes moderating factors in the relationships between causes and axial categories, axial categories and outcomes, and axial categories and moderating factors. In this study, contingencies are the demographic and behavioural characteristics of leaders, followers, and departments that moderate (i) the relationship between the axial category and mediating conditions, and (ii) the relationship between the axial category and outcomes by affecting the followers’ interpretation and perception of leader humour. In addition, the aforementioned contingencies moderate (iii) the relationship between the causes and the axial category by improving the conversion of leader sense of humour into humorous behaviour. Regarding the relationship between sense of humour and humorous behaviour in this study, the introduced moderating role points to the characteristics of the leader, followers, and departments that would help the leader to adopt a sense of humour more easily. According to the data analysis of this study, demographics of leader and followers refer to their literacy, career, and age similarity of leaders with followers; demographics of the department refer to the department size and composition. The behavioural and personality characteristics of the followers include followers’ sense of humour, previous experience, follower insight into the leader, cynicism, humility, sensitive personality, prejudice, and malice. The results of the study show that demographics and behavioural characteristics of followers refer to the factors that affect their perception and interpretation of the leader’s humorous behaviour and occurrence of possible future resentment in relationships; this therefore encourages the leader to perform humorous behaviours and increases the possibility of converting sense of humour into humorous behaviour (thorough the mediating conditions). The behavioural and personality characteristics of the leaders include justice, charisma, humility, lack of explicitness, and hypocrisy. As a result of the interview analysis, the demographic and behavioural characteristics of leaders make them better understand the conditions of converting sense of humour into behaviour, while reducing the possibility of misunderstanding their humour, increasing its positive outcomes. For instance, modest leaders do not consider the right and appropriate humour to be against their dignity and position, and as a result, express their sense of humour if necessary. The similarity of the leader’s age with the followers makes it easier for the leader to express his sense of humour; moreover, the lack of explicitness and hypocrisy probably makes the leader’s humour acceptable and helps prevent misunderstanding, thus increasing its positive outcomes. Department demographics refer to department size and composition and behavioural characteristics refer to the department cohesion, norms, and the level of acceptance of the individual by group. This study’s findings suggest that the size of the group (department) and its composition (in age and gender), alongside the level of acceptance of individuals by the department, affect the conditions for the leader to express his sense of humour as well as the followers’ perceptions of the leader’s humour. The chances of successful humorous behaviour are higher when leaders and followers are similar in terms of age and gender, and know each other well. Wisse and Rietzschel [70] also proved the existence of such a relationship when the humour styles of leaders and followers are similar. Consistent with the results of this study, Tan et al. [22] demonstrated the moderating role of gender differences between leaders and followers in empowering the reverse relationship between traditionalism and the expression of leader humour. The results of this study suggest that group norms also cause the leader to express humorous behaviour or suppress it, and have a great impact on people’s perception of behaviours. Group cohesion improves the conditions for the leader to express his sense of humour; it also prevents misunderstanding about his humorous behaviour and brings many positive outcomes.

However, the moderating relationships of humorous behaviour with mediating factors and outcomes indicate the factors affecting followers’ perceptions and interpretations of leaders’ humorous behaviour. Specifically, the presence of certain demographic and behavioural characteristics of leaders, followers, and departments can cause differences in perceptions of leader humorous behaviour. Martin and Ford [2] stated that reaction to humour (and its interpretation) depends on the status, context, and position of the humourist relative to the listener. Moreover, different reactions to humour might be due to differences in role expectations that people have of each other [2]. Decker and Rotondo [71] and Moake and Robert [72] indicated that the gender of followers and leaders affects perceptions of leader humour. Priest and Swain [44] conducted two studies in the military to evaluate the hypothesis that leaders are considered ‘good’ or ‘bad’ based on perceptions of their sense of humour. They asked participants to think about a ‘good’ or ‘bad’ leader. In contrast to bad leaders, good leaders were described as having warm, competent, and benign humorous styles. For instance, the participants stated that a good leader ‘uses good-natured jest to put others at ease’ and that ‘bad’ leaders made fun of naïve and simple people and were unable to laugh at their failings [44]. Wisse and Rietzschel [70] concluded that the perception of leader humour style is more important in predicting the quality of relationships than the actual style of humour.

In line with this study, Robert et al. [60] found that followers’ job satisfaction is affected by the perceived quality of leader–follower relationships, but has nothing to do with humour style, and is not affected by a leader’s use of affiliative or aggressive humour. They argued that followers are more likely to consider leaders’ humour positive when they evaluate their relationships with leaders positively (regardless of the style used by the leaders). Simultaneously, a negative relationship can make employees consider leader humour as relatively negative, even when leaders have positive intentions [60]. Under such conditions, employees may view the use of humour as inappropriate or that it only serves as a distraction or a potential tactic for ingratiation [45]. Therefore, humour outcomes are based more on the relationship between leaders and followers and not on the type of humour [60]. Scheel and Gockel [45] argued that (the consequences of) leader humour depend more on listeners’ ears (perception) than on speakers’ mouths (type of humour).

Although the results of this study are consistent with some of the above findings, a new finding is introduced, namely, that the outcomes of leader humour depend on the demographic and behavioural characteristics of listeners (i.e., the intrinsic factor of interpretation) as well as the demographic and behavioural characteristics of speakers (indicating leader’s intention) and those of departments (quality of relationships and norms of departments). Specifically, these characteristics form the interpretations and perceptions. For instance, as a result of the interview analysis, a leader’s humility, followers’ sense of humour, and followers’ insights into the leader minimise the misunderstanding of their humorous behaviour because such a leader’s humorous behaviour does not usually involve any signs of mockery or humiliation of others. Thus, such a leader is not worried about other people misunderstanding their behaviour and can express their sense of humour more easily. The modest humour style introduced by Martin et al. [49] presents a concept similar to humility (self-deprecating); however, it differs from humility introduced in this section. Meanwhile, the results of the interview analysis showed that a leader’s hypocrisy (noticed by followers) or the sensitive spirit or malice of the followers can also lead to a misinterpretation of their positive humour. In addition, followers’ cynicism under any conditions can cause false perceptions of leader humour, whereas the additive norms of departments can cause false positives of leader humour.

Therefore, the most important identified component is followers’ sense of humour. This study’s findings show that followers’ sense of humour is the most important factor in their perception and interpretation of leader humorous behaviour, a finding that has not been extensively explored in prior studies. High levels of humour in followers indicate high levels of capacity, intelligence, and perception of their situations, which result in a better perception and acceptance of leader humour and no misinterpretations of their behaviour; leaders are more likely to express humorous behaviour when they observe that their followers have a sense of humour. However, factors such as prejudice, malice, and lack of humility could prevent them from properly using their sense of humour, thereby causing misinterpretation. To our knowledge, this study is one of the first studies to determine the demographic and behavioural characteristics of leaders, followers, and departments as the moderator of relationships between causes (sense of humour) and the axial category (humorous behaviour), between the axial category and outcomes, and between the axial category and mediating factors. Moreover, paying attention to the moderating effect of “follower sense of humour” is of special importance due to attraction and coordination with previous theories (e.g., incongruity [38]), comprehension-elaboration [73], and benign violation [36], including recent findings on humour intelligence [47]. High levels of follower sense of humour can help them better understand the sweet point, perceive a violation as benign and harmless, and minimise the chances of misinterpretation.

### Limitations

The nature of the research topic is one of the limitations of this study, because leaders, like any other person, may not accurately evaluate their sense of humour, and followers may sometimes exhibit unrealistic reactions to leaders’ humour expressions. It is therefore preferable to collect and analyse leaders’ sense of humour from their perspective and evaluate the success and appreciation of their humorous behaviour from the perspective of followers. This requires the collection of a great deal of information from various departments, chairs, and all members, which requires substantial time, money, and effort. Additionally, the final model based on the GT method involves various relationships whose quantitative testing in a single study appears practically impossible.

### Suggestions for future studies

Considering that this is a qualitative study, the relationships of its components are analysed quantitatively. Therefore, it is recommended to analyse the importance of extremism and moderation in leader humour quantitatively based on TMGT theory [44] to confirm (or unlikely reject) the probability of any curvilinear relationship. Consistent with many other researchers, this study viewed sense of humour as a transcendental phenomenon, which would probably have no reverse relationship with positive outcomes. However, in the best case, humour requires moderation. Violations of moderation (repetition and alteration of humour) can cause the same destructive outcomes as non-normative humour and offensive behaviour accompanied by laughter. Researchers should analyse this probability in future studies.

Determining the components of leader sense of humour in this study can help develop new ways to measure this construct. The existing measures of sense of humour tend to have low internal consistency and the components of sense of humour and how they are interrelated require further conceptual and empirical work [18]. According to the proposed model, a new questionnaire should be developed and validated to measure the different aspects of this transcendental attribute.

Paying attention to follower sense of humour (and leader sense of humour) in this study can be considered a response to the invitation made by Cooper et al. [41]. Hence, it is advisable to measure quantitatively the concurrent effects of leader sense of humour and follower sense of humour. Future studies can also evaluate the humour expressed by the followers of leaders, or simultaneously analyse the humour expressions of both followers and leaders. Therefore, the effects will be differentiated, and it will be possible to measure humour, which is more important in terms of quality exchange relationships and the induction of positive emotions and outcomes.

Given the emphasis of this study and other studies on the necessity of paying attention to the context and culture in which humour manifests, is ‘sense of humour’, like ‘humour’, a series of attributes and components based on context? Does each context require a specific sense of humour? Conducting similar studies in other contexts and comparing their results with the findings of this study could answer these questions.

Since norms are generally based on the contexts and perceptions of people present in a specific situation, it is important to consider context in studies of the sense of humour. However, a more important question is how leader humour can lead to different interpretations and reactions on the part of the audience within the same context. We call this ‘gray humour’ as it is neither completely benign nor aggressive. In this study, individual differences, behavioural characteristics, and the personality traits of leaders and followers were introduced as factors that cause differences in the interpretation of humour. A quantitative analysis of the moderating effects of ethical characteristics, such as suspicion (among followers) and humility (among leaders), should be conducted based on data collected from interviewers concerning the factors affecting the interpretation of humour (contingencies).

## Conclusion

Researchers who have reviewed the literature (e.g [18, 41]), concluded that ambiguities, gaps, challenges, and contradictions still exist regarding the conceptualization and clarity of humour expression and sense of humour. This study aimed to address some of these gaps and ambiguities. One notable finding is the distinction between leaders’ sense of humour and their humorous behaviour. Moreover, the study identifies sense of humour as the cause of a leader’s humorous behaviour. The elements of a leader’s sense of humour include capacity, situational perception, creativity, emotional intelligence, verbal intelligence, and individual differences.

Based on our review of the literature, we did not find any studies that specifically focused on separating and explaining elements of leaders’ sense of humour as the causes of humorous behaviours. This study categorises intelligence into verbal intelligence and emotional intelligence. Another innovative aspect of this research is the inclusion of both followers’ and leaders’ sense of humour, along with their personal and behavioural traits. The study uniquely addresses the importance of moderation (and avoidance of extremism) in positive humour with benevolent intentions. It also identifies leader, follower, and group demographics as moderators influencing the expression of humour based on sense of humour, as well as mediating conditions and the outcomes of leader humour. The literature review found no prior research addressing most of these components and their correlations.

Therefore, this study expands and develops the theoretical foundations of sense of humour. Regarding the emerging factors and components, followers’ sense of humour (as a moderator) and the necessity of paying attention to moderation in a leader’s humour expression in the model for leaders’ sense of humour can be considered the main innovation of this study and the factors of its uniqueness. Furthermore, Kong et al. [18] conducted a meta-analysis and concluded that the vast majority of studies on leaders’ sense of humour either focused on the main effects of leader trait humour or leader humour expression; very few studies examined moderators for the effects of leader humour or the moderating effect of leader humour, without the guidance of a systematic framework. Therefore, they invite researchers to pay more attention to the moderators of leaders’ humour outcomes with a more comprehensive look. Adopting a GT approach, this study takes a universal look not only to determine the moderators of leaders’ humorous behaviour outcomes but also to discover their role in moderating other relationships (between the components of sense of humour and leader humorous behaviour as well as between humorous behaviour and mediating conditions).

Studies of leadership (and qualitative studies in general) tend to be context-based, and their approaches vary based on the prevailing culture of societies and countries. Therefore, implementing this study in Iran, a developing country, especially in an academic and higher-education environment ecosystem, can be considered another research innovation. The ever-increasing growth in studies of leaders’ sense of humour has led to a better understanding of this valuable concept and its practical aspects. However, there is still no simple and clear path for its practical application. There may never be any simple recipe and guidelines for leaders on how to use humour correctly [45].

This study explains the causes, correlated causes, mediators, moderators, contexts, and consequences of leaders’ humorous behaviours in general and those of academic department chairs in particular, concerning the various aspects of this inevitable phenomenon. Before practical suggestions are made, two points must be taken into account. First, sense of humour (closely linked to intelligence) is an innate characteristic rather than an acquired one. Specifically, individuals must have an average sense of humour for it to be nurtured and developed. Morose individuals cannot be made humorous; however, sense of humour can be improved in people, and people can be made familiar with the correct and effective methods of using a sense of humour. Moreover, a sense of humour can never replace the efficiency and effectiveness of leaders or be considered the sole and unique tool for achieving joy and happiness in a workplace. It is merely one of the effective communication tools leaders use to stimulate positive emotions among followers. Before expressing humour, leaders should thoroughly consider their relationships with their followers and followers’ sense of humour and other characteristics. Negative relationships, a low level of sense of humour in followers, and excessive humour on the part of leaders, may lead to misunderstandings about humour among followers, even if leaders pursue positive goals and benevolent intentions. In such situations, leaders should create the necessary base to provide a more conducive environment.

This study’s results provide a new, comprehensive understanding of leader humour. Educational department chairs typically have no control over financial and economic resources. Humour is considered a socioemotional resource [4] that while not economically cost-effective, is highly valuable for communication. Hence, the findings can help improve the understanding of department chairs.

## Data Availability

Study data are accessible at formal request and with the permission of the authorities of the Ferdowsi University of Mashhad, Iran.
